# Psychiatric Symptoms and Frequency of Eating out among Commuters in Beijing: A Bidirectional Association?

**DOI:** 10.3390/nu14204221

**Published:** 2022-10-11

**Authors:** Ling Zhang, Yunyi Xie, Bingxiao Li, Fuyuan Weng, Fengxu Zhang, Juan Xia

**Affiliations:** 1 Department of Epidemiology and Health Statistics, School of Public Health, Capital Medical University, Beijing 100069, China; 2 Beijing Municipal Key Laboratory of Clinical Epidemiology, Capital Medical University, Beijing 100069, China

**Keywords:** eating out, depression, stress, anxiety, commuters

## Abstract

Background: Mental illness places as a distant first in global burdens, exceeding both cardiovascular and circulatory diseases, in terms of the years lived with the disability. The emergence of the new and burgeoning area of “Nutrition Psychiatry” offers promise in improving mental health with diet. Mental health and well-being are critical to commuters but rarely recieve the attention they need. This study aimed to examine the bidirectional relationship between the frequency of eating out and depression, anxiety, and stress symptoms in a sample of Beijing commuters. Methods: A total of 3337 commuters (mean (SD) age, 38.78 (10.41); 74.74% males) from the cohort study CHCN-BTH were included. The psychiatric symptoms were evaluated using a 21-item self-reported depression–anxiety–stress scale (DASS-21). A Cochran–Armitage trend chi-square test, restricted cubic spline, multiple logistic regression, multinomial logit models, and E-values were performed to estimate the associations between eating out and psychiatric symptoms in both directions. Results: A daily rate of eating out more than 50% had a higher risk for depression (OR, 95% CI: 1.68, 1.184–2.393), anxiety (1.73, 1.259–2.369), and stress (1.99, 1.191–3.329) than the individuals eating at home. A higher frequency of eating out for lunch was significantly associated with an increased risk of depression (1.78, 1.28–2.46), anxiety (1.67, 1.26–2.23), and stress (2.05, 1.31–3.22). Similar results were found when eating out for dinner with increased risks for depression 2.20 (1.59, 3.06), anxiety 1.91 (1.42, 2.59), and stress 2.61 (1.68, 4.05). There is limited evidence supporting the effects of psychiatric symptoms on the frequency of eating out in the reverse analyses. Conclusions: The frequency of eating out is positively associated with an increased risk of psychiatric symptoms, especially when eating out for lunch and dinner. People eating at home have the lowest risk of suffering psychiatric symptoms, followed by those eating in the workplace canteen. Eating at home should be considered for future recommendations for the prevention of psychiatric symptoms.

## 1. Introduction

The popularity of eating outside of the home (takeaway, take out, and fast food) has increased exponentially in recent decades and is thought to be a key driver in increasing levels of negative health outcomes due to the high quantities of unhealthy ingredients in this food. Individual food choices and eating behaviors are influenced by many interrelated factors, such as societal, environmental, psychological spheres [1]. Previous evidence has demonstrated that eating outside of the home tends to involve bigger portion sizes and unhealthier food choices compared with eating at home. Additionally, food from outside the home tends to be more energy dense and contain more total fat, saturated fat, sodium, and sugar than foods prepared at home [2,3,4,5,6]. These types of food are thought to be linked to a higher risk of obesity, chronic diseases, and all-cause and cause-specific mortality [7,8,9]. However, little is known about the relationship between eating outside of the home and the risk of depression, anxiety, and stress symptoms among commuters.

Mental health and well-being are critical to commuters, but they rarely recieve the attention they need. Workers have been seeing increases in mental illness and emotional distress and the COVID-19 pandemic and its social and economic consequences are accelerating these trends [10,11]. The previous results reported that the prevalence of moderate-to-severe stress, anxiety and depression in the general Chinese population were 8.1%, 28.8%, and 16.5%, respectively, during the COVID-19 epidemic [12]. While the proportions were higher among health care workers: depression (50.4%), anxiety (44.6%), and stress (71.5%) [13]. Mental illness can lead to many negative health outcomes such as quality of life [14], suicide [15], physical diseases, and mortality [16]. Promoting mental health is important for human health, after all, physically and mentally healthy people are what society really needs.

The emergence of the new and burgeoning area of “Nutrition Psychiatry” offers promise in improving mental health using diet [17,18,19]. The quality of people’s meals usually varies depending on where they eat, therefore, we aimed to examine the relationship between the frequency of eating outside of the home with depression, anxiety, and stress symptoms in a sample of Beijing commuters based on data from a cohort study on chronic disease of communities natural population in Beijing, Tianjin, and Hebei (CHCN-BTH).

## 2. Materials and Methods

### 2.1. Participants and Data Collection

Data used in this study were obtained from the CHCN-BTH, which was approved by the Ethics Committee of the Centre of Disease Control (IRB2017-003, CYCDPCIRB-20170830-1) and Capital Medical University (2018SY81). Additionally, this study was registered in the Chinese Clinical Trial Registry (http://www.chictr.org.cn/showproj.aspx?proj=26656, ChiCTR1900024725, accessed on 25 July 2019). The design and rationale of the study have been described previously in detail [20,21]. Signed informed consent was obtained from all individuals prior to the survey. All the data were collected through a face-to-face personal interviews under the guidance of trained researchers, and all interviewers completed unified training before they participated in the formal survey. The subjects of this study were mainly office workers in public institutions, including management staff and technical staff. A total of 3337 commuters with complete dietary and mental health assessment information at the time of follow-up from July to September 2021 were included in the present analysis. We excluded participants with missing information regarding depression, anxiety, or stress symptoms, resulting in a final analysis sample of 3313 commuters (Supplemental Figure S1).

### 2.2. Depression, Anxiety, and Stress Symptoms

The 21-item self-reported depression–anxiety–stress scale (DASS-21) was used to evaluate the participants’ depression, anxiety, and stress symptoms (dependent variable). The scale was developed using Australian data and has been validated in both clinical [22] and nonclinical samples [23], in the community [24], and with adults [25]. The DASS-21 possessed good convergent and discriminant validity and high internal consistency in our population also. The cut-off scores of greater than 8, 6, and 13 represent a positive screen of depression, anxiety, and stress, respectively [26].

### 2.3. Frequency of Eating out 

The frequency of eating out was explored to determine its association with mental health. Participants were asked to report how many days in a week they were eating out (including at a work canteen) for breakfast, lunch, and dinner. Responses were divided into four groups: “0 times a week”, “1–4 times per week”, “5 times per week” and “more than 5 times per week”. The daily rate of eating out was calculated as a daily eating out frequency × 100%/daily eating frequency, and stratified as “none (0%)”, “less than 50%”, and “more than 50%”.

### 2.4. Covariates

The following potential confounders were selected based on previous research: age groups (divided into four groups according to the quartile), gender, marital status (divided into two categories based on four categories in the questionnaire), education status (grouped into three levels: senior secondary, university or college, or postgraduate and above), smoking status (never, previous smoker, or current smoker), alcohol drinking status (never, previous drinker, or current drinker), physical exercise (5–7, 1–4, or <1 days per week), chronic diseases (yes or no), and body mass index (BMI, calculated as weight in kilograms divided by the square of height in meters). BMI was divided into four groups (underweight: <18.5 kg/m^2^, normal: 18.5–23.9 kg/m^2^, overweight: 24.0–27.9 kg/m^2^, and obesity: >28.0 kg/m^2^) according to the criteria recommended in China [27]. 

### 2.5. Statistical Analysis

Continuous variables were summarized with the mean and standard deviation (SD), and categorical variables were described as the count and percentage. Trends across ordered categories of frequency of eating out were tested using the Cochran–Armitage trend chi-square test. Associations between the frequency of eating out for breakfast, lunch, and dinner with depression, anxiety, and stress were assessed using multiple logistic regression models. Restricted cubic spline analyses were performed to estimate the associations between rates of eating out (on a continuous scale) and the risk of depression, anxiety, and stress. The multinomial logit models were used to estimate the associations between psychiatric symptoms and the frequency of eating out. All statistical analyses were performed in SAS 9.4 (SAS Institute, Inc., Cary, NC, USA), and visualization of the results was implemented in GraphPad Prism version 9 (https://www.graphpad.com, accessed on 21 September 2022). A two-side *p*-value of <0.05 was considered statistically significant. Reporting of the study conforms to broad EQUATOR guidelines [28].

### 2.6. Sensitivity Analyses

Additional sensitivity analysis for the potential effect of unmeasured confounders was performed by the E-value methodology of VanderWeele and Ding [29,30]. This method estimates the minimum strength of association that would be required between an unmeasured confounder and both eating out and psychiatric symptoms to overcome the statistically significant effect observed in this study [29]. The calculation is derived from the odds ratios obtained from an adjusted analysis in observational studies.

## 3. Results

### 3.1. Characteristics of the Study Population

The final analysis included 2494 males (mean [SD] age, 39.4 [10.92] years) and 843 females (mean [SD] age, 36.95 [8.47] years). The prevalence of depression, anxiety, and stress were 14.40%, 19.63%, and 6.70%, respectively. Five hundred and forty-one commuters (16.32%) reported a daily rate of eating out of 0%, 1669 commuters (50.36%) reported a daily rate of eating out of less than 50%, and 1104 commuters (33.31%) reported a daily rate of eating out of higher than 50%. The frequency of eating out in males was significantly higher than females, regardless of whether it was for breakfast, lunch, or dinner. Table 1 presents the characteristics of 3337 participants reported by gender.

### 3.2. Distribution of Depression, Anxiety, and Stress according to the Frequency of Eating out

The prevalence of depression, anxiety, and stress suffered by commuters were all increased with the frequency of eating away from home, especially for lunch and dinner (Table 2). People who ate out for breakfast more than five times per week had a 3.36%, 7.24%, and 3.48% higher prevalence of depression, anxiety, and stress, respectively, than those who ate at home. For lunch, people who ate out more than five times per week had a 7.75%, 9.70%, and 5.65% higher prevalence of depression, anxiety, and stress, respectively, than those who ate at home. For dinner, people who ate out more than five times per week had a 10.87%, 11.66%, and 7.01% higher prevalence of depression, anxiety, and stress, respectively, than those who ate at home.

### 3.3. Associations between Frequency of Eating out and Mental Health

**Figure 1:**
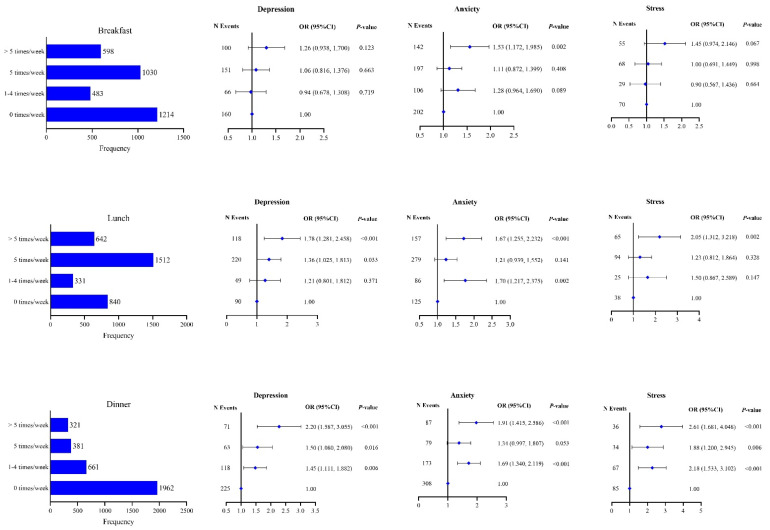
Associations between mental health and frequency of eating out among commuters in Beijing. Adjusted for age, gender, marital status, education level, smoking, drinking, physical exercise, BMI, and chronic diseases.

Figure 1 presents the results of multiple logistic regressions testing the associations between the frequency of eating out for breakfast, lunch, and dinner with mental health after controlling for demographic characteristics, with “eating at home” as the referent group. Comparing the participants who ate out more than five times per week with those eating at home, the multivariable-adjusted ORs (95% CI) of depression were 1.26 (0.94, 1.70) for breakfast, 1.78 (1.28, 2.46) for lunch, and 2.20 (1.59, 3.06) for dinner; the multivariable-adjusted ORs (95% CI) of anxiety were 1.53 (1.17, 1.99) for breakfast, 1.67 (1.26, 2.23) for lunch, and 1.91 (1.42, 2.59) for dinner; and the multivariable-adjusted ORs (95% CI) of stress were 1.45 (0.97, 2.15) for breakfast, 2.05 (1.31, 3.22) for lunch, and 2.61 (1.68, 4.05) for dinner.

**Figure 2:**
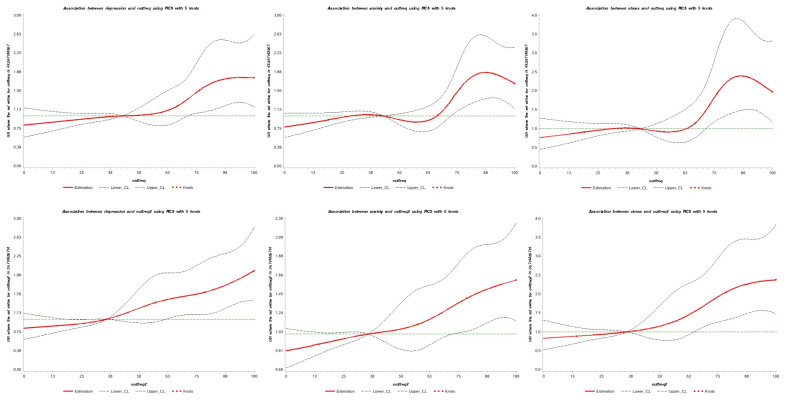
Restricted cubic spline model of the odds ratios of mental health with the frequency of eating out. Adjusted for age, gender, marital status, education level, smoking, drinking, physical exercise, BMI, and chronic diseases. The daily rate of eating out for the graphs in the first row was calculated with breakfast, lunch, and dinner; the second row was calculated with lunch and dinner.

Restricted cubic spline analysis of the associations between rates of eating out (on a continuous scale) and the risk of depression, anxiety, and stress for all participants showed an increasing risk of suffered mental illness with increased rates of eating out at different levels (Figure 2). In particular, the rates of eating out for lunch and dinner demonstrated a linear increase with mental illness.

### 3.4. Associations between Psychiatric Symptoms and the Frequency of Eating Out

Table 3 demonstrates the results of multinomial logit model testing the associations between psychiatrics symptoms with the frequency of eating out. The results indicated that none of the three psychiatric symptoms were associated with eating out. The adjusted ORs of eating out more than 50% were 1.21 (95% CI: 0.78–1.90; *p* = 0.396) for depression, 1.46 (95% CI: 0.99–2.15; *p* = 0.056) for anxiety and 1.34 (95% CI: 0.73–2.46; *p* = 0.342) for stress.

### 3.5. Sensitivity Analysis for Unmeasured Confounders

The E-values for the point estimate and upper confidence interval limit at the daily rate of eating out more than 50% were 2.75 and 1.65 for depression, 1.96 and 1.49 for anxiety, and 3.39 and 1.67 for stress. For the higher frequency of eating out for lunch, the E-values for the point estimate and upper confidence bound for depression, anxiety, and stress were 2.96 and 1.88, 1.91 and 1.49, and 3.52 and 1.95, respectively. The E-values for the point estimate and upper confidence interval limit at the higher frequency of eating out for dinner were 3.82 and 2.56 for depression, 2.11 and 1.67 for anxiety, and 4.66 and 2.75 for stress, and at the higher frequency of eating out for breakfast they were 1.83 and 1 for depression, 1.78 and 1.38 for anxiety, and 2.26 and 1 for stress.

## 4. Discussion

This study has shown that the prevalence of depression, anxiety, and stress symptoms were 14.40%, 19.63%, and 6.70% among commuters in Beijing, respectively, which is consistent with previous studies [12,31,32], although these are now slightly lower than the levels at the beginning of the COVID-19 outbreak. This is concerning because the poor mental health of workers can not only create significant problems in the workplace and reduce work efficiency and productivity [33,34,35], but it may also increase negative health outcomes both for the workers themselves [36,37] and their children [38]. The results indicated that the frequency of eating out were associated with an increased risk of depression, anxiety, and stress symptoms, especially when eating out for lunch and dinner. 

Previous studies have reported that eating outside of the home has been linked to increased consumption of higher calories, as well as a higher intake of saturated or total fat, sugars, and sodium [39], but a lower consumption of whole grains, vegetables, fruit, and protective nutrients such as dietary fibers, micronutrients (particularly vitamin C, Ca, and Fe), and antioxidants [6]. The emerging evidence examining the relationship between diet and psychiatric symptoms has demonstrated that diets characterized by a high consumption of whole grains, fruit, vegetables, nuts, fish, and with limited processed foods were inversely associated with the risk for depression [40]. In contrast, diets characterized by a high intake of fat, sugar and high in processed foods were positively associated with mental disorders [40]. This study found that those individuals who had a daily rate of eating out of more than 50% had a higher risk of depression (1.68, 1.184–2.393; *p* = 0.004), anxiety (1.73, 1.259–2.369; *p* < 0.001), and stress (1.99, 1.191–3.329; *p* = 0.009) than individuals who ate at home. However, the inverse analysis results showed that none of the three psychiatric symptoms were associated with eating out, which indicated that the reverse hypothesis that those who were more likely to be anxious, depressed, or stress may be more likely to eat out to leave their homes and not be alone was invalidated in this study.

For breakfast, significant association between the frequency of eating out and psychiatric symptoms were only found in anxiety symptoms in this study. Standing in line to buy breakfast when eating outside of the home or being in a rush to avoid being late for work may cause commuters to have increased anxiety symptoms. There is usually a wider variety of food choices for lunch and dinner than for breakfast, which may result in little difference for eating breakfast at or out of the home. Furthermore, people usually eat food with less energy and of a poorer variety at breakfast than at lunch or dinner. Those might be some of the reasons for the insignificant association between the frequency of eating out for breakfast with depression and stress. For lunch and dinner, the group who were eating outside of their home five times per week were regularly eating meals in their workplace canteen, and the food in the canteen was relatively healthier than takeaway. This may be a reasons why the effects of eating outside of the home five times per week on psychiatric symptoms were lower than the groups who ate outside of their home of 1–4 times per week or more than five times per week.

The mechanisms of nutritional impact on mental health are compound and complex. Current research has focused on the hippocampal neurogenesis which is the brain region associated with cognition and mood and is one of two structures where neurogenesis persists [41]. Therefore, modulation of the hippocampal neurogenesis through diet emerged as a possible mechanism for the effects of nutrition on mental health [42]. Evidence on dietary interventions and putative mechanisms of action is being studied and remains to be further explored in this field. Changing the eating out behavior of people and eating at home whenever possible so as to improve the quality of their meals is important for reducing the risk of suffering psychiatric symptoms.

A recent study reported that the global burden of mental illness accounts for 32.4% of years lived with disability and 13.0% of disability-adjusted life-years, and the approaches used may underestimate the burden of mental illness by more than a third. It has been estimated that mental illness places as a distant first in global burdens, in terms of years lived with disability, exceeding both cardiovascular and circulatory diseases [43]. Additionally, evidence from a Chinese study poses serious challenges related to the high burdens of mental illness identified [44]. Due to the magnitude of the psychiatric symptom burden, and the universality of food and eating behaviors as modifiable risk factors, even small improvements in the nutritional environment can translate into dramatic improvements in mental health and well-being at a population level. 

This study focused on the associations between eating behavior and mental health among commuters in Beijing. The strengths of this study include using both restricted cubic spline and multiple logistic regression models to analyze the results, and performing a separate analysis of the relationship between the frequency of eating out for breakfast, lunch, and dinner with psychiatric symptoms (depression, anxiety, and stress), which strengthened the conclusions. However, several limitations should be noted. First, the frequency of eating outside of the home was self-reported and did not distinguish between the quality of foods consumed, fast food, or healthier options. Participants who reported eating out more often may chose healthier options at dine-out restaurants or fast-food chains. However, results from the food consumption in this study showed that eating out was linked to a high intake of meats and fried foods and a lower intake of vegetables, fruit, and milk, which may attenuate the impact of the results. Second, despite controlling for some confounding factors, the possibility of residual and unmeasured confounders cannot be completely ruled out due to the observational nature of the study. The distance from the residence to the workplace, family relationships, the family environment, time availability, the quality of outside catering, etc., certainly play a certain role on eating outside the home. However, the sensitivity analysis using E-value methodology indicated that the observed ORs for psychiatric symptoms could only be explained by an unmeasured confounder that was associated with both eating out and the risk of psychiatric symptoms by an odds ratio of more than 2.75 (depression), 1.96 (anxiety), and 3.39 (stress) above and beyond that of the confounders that were measured in the present study. Given that these ORs were much greater than the observed for the known risk factors of the psychiatric symptoms examined in this study, it was implausible that an unmeasured confounder existed that could overcome the effect of eating out observed in the present study. Third, the generalizability of the findings may be limited because participants in this study were predominantly commuters in Beijing. Fourth, the rate of eating out may be underestimated due to the impact of the COVID-19 pandemic, although the epidemic was well under control in our country when we conducted the survey. Further studies are needed to confirm the association between eating behavior and the frequency of eating out in other populations in regions of different economic levels.

## 5. Conclusions

There have been some advances in our understanding of the association between eating behavior and mental health. This study found that the frequency of eating out was positively associated with an increased risk of depression, anxiety, and stress symptoms, especially for the frequency of eating out for lunch and dinner. People eating at home had the lowest risk of suffered psychiatric symptoms, followed by those eating in the workplace canteen. However, there was limited evidence supporting the effects of psychiatric symptoms on the frequency of eating out in the reverse analyses. Future studies are needed to confirm the associations from other populations in regions of different economic levels. Eating at home could be considered for future recommendations for the prevention of psychiatric symptoms, although more evidence is needed.

## Figures and Tables

**Table 1:** Characteristics of the participants by gender.

	Total Sample	Male	Female
Total sample, *n* (%)	3337 (100%)	2494 (74.74%)	843 (25.26%)
Age (years), mean ± SD	38.78 ± 10.41	39.40 ± 10.92	36.95 ± 8.47
Education			
Senior secondary	864 (25.89)	712 (28.55)	152 (18.03)
University or college	2010 (60.23)	1502 (60.22)	508 (60.26)
Postgraduate and above	463 (13.87)	280 (11.23)	183 (21.71)
Marriage			
Single	723 (21.67)	558 (22.37)	165 (19.57)
Married	2614 (78.33)	1936 (77.63)	678 (80.43)
Smoking			
Never a smoker	1986 (59.69)	1157 (46.54)	829 (98.57)
Previous smoker	206 (6.19)	204 (8.21)	2 (0.24)
Current smoker	1135 (34.11)	1125 (42.25)	10 (1.19)
Drinking			
Never a drinker	1478 (44.38)	764 (30.71)	714 (84.80)
Previous drinker	124 (3.72)	120 (4.82)	4 (0.48)
Current drinker	1728 (51.89)	1604 (64.47)	124 (14.73)
Exercise			
5–7 d/w	727 (21.79)	600 (24.06)	127 (15.07)
1–4 d/w	1233 (36.95)	927 (37.17)	306 (36.30)
<1 d/w	1377 (41.26)	967 (38.77)	410 (48.64)
BMI			
Underweight	78 (2.50)	31 (1.32)	47 (6.14)
Normal	1184 (37.96)	723 (30.73)	461 (60.18)
Overweight	1197 (38.38)	1019 (43.31)	178 (23.24)
Obesity	660 (21.16)	580 (24.65)	80 (10.44)
Frequency of eating out for breakfast			
0 times per week	1207 (36.42)	798 (32.22)	409 (48.86)
1–4 times per week	483 (14.57)	372 (15.02)	111 (13.26)
5 times per week	1028 (31.02)	815 (32.90)	213 (25.45)
>5 times per week	596 (17.98)	492 (19.86)	104 (12.43)
Frequency of eating out for lunch			
0 times per week	836 (25.23)	564 (22.77)	272 (32.50)
1–4 times per week	331 (9.99)	237 (9.57)	94 (11.23)
5 times per week	1507 (45.47)	1158 (46.75)	349 (41.70)
>5 times per week	640 (19.31)	518 (20.91)	122 (14.58)
Frequency of eating out for dinner			
0 times per week	1955 (58.99)	1402 (56.60)	553 (66.07)
1–4 times per week	659 (19.89)	499 (20.15)	160 (19.12)
5 times per week	380 (11.47)	301 (12.15)	79 (9.44)
>5 times per week	320 (9.66)	275 (11.10)	45 (5.38)
Rate of eating out			
0%	541 (16.32)	343 (13.85)	198 (23.66)
1–50%	1669 (50.36)	1234 (49.82)	435 (51.97)
51–100%	1104 (33.31)	900 (36.33)	204 (24.37)
Chronic diseases	748 (22.47)	635 (25.51)	113 (13.45)
Depression	477 (14.40)	348 (14.06)	129 (15.41)
Anxiety	650 (19.63)	469 (18.95)	181 (21.62)
Stress	222 (6.70)	173 (6.99)	49 (5.85)

Data are percentages unless otherwise indicated.

**Table 2:** Distribution of depression, anxiety, and stress by the frequency of eating out.

	Depression	Anxiety	Stress
Frequency of eating out for breakfast			
0 times per week	160 (13.23)	202 (16.71)	70 (5.79)
1–4 times per week	66 (13.72)	106 (22.04)	29 (6.03)
5 times per week	151 (14.76)	197 (19.26)	68 (6.65)
>5 times per week	100 (16.86)	142 (23.95)	55 (9.27)
$\;\chi^{2}$ (*P*)	−2.027 (0.043)	−3.201 (0.001)	−2.489 (0.013)
Frequency of eating out for lunch			
0 times per week	90 (10.77)	125 (14.95)	38 (4.55)
1–4 times per week	49 (14.80)	86 (25.98)	25 (7.55)
5 times per week	220 (14.65)	279 (18.58)	94 (6.26)
>5 times per week	118 (18.52)	157 (24.65)	65 (10.20)
$\;\chi^{2}$ (*P*)	−4.00 (<0.001)	−3.637 (<0.001)	−3.637 (<0.001)
Frequency of eating out for dinner			
0 times per week	225 (11.53)	308 (15.78)	85 (4.35)
1–4 times per week	118 (17.93)	173 (26.29)	67 (10.18)
5 times per week	63 (16.62)	79 (20.84)	34 (8.97)
>5 times per week	71 (22.40)	87 (27.44)	36 (11.36)
$\;\chi^{2}$ (*P*)	−5.693 (<0.001)	−5.622 (<0.001)	−5.818 (<0.001)
Rate of eating out			
0%	56 (10.37)	75 (13.89)	22 (4.07)
1–50%	222 (13.30)	310 (18.57)	94 (5.63)
51–100%	199 (18.14)	262 (23.88)	106 (9.66)
$\;\chi^{2}$ (*P*)	−4.538 (<0.001)	−5.004 (<0.001)	−4.739 (<0.001)

Results reflect a two-tailed significance test from Cochran–Armitage trend chi-square test.

**Table 3:** Associations between psychiatric symptoms and the frequency of eating out among commuters in Beijing.

Psychiatric Symptoms	1–50%		51–100%	
	No.	OR (95% CI)	No.	OR (95% CI)
Depression				
No	1447	ref	898	ref
Yes	222	1.01 (0.662, 1.528)	199	1.21 (0.776, 1.897)
Anxiety				
No	1359	ref	835	ref
Yes	310	1.32 (0.920, 1.891)	262	1.46 (0.991, 2.152)
Stress				
No	1575	ref	991	ref
Yes	94	0.98 (0.544, 1.755)	106	1.34 (0.732, 2.463)

Adjusted for age, gender, marital status, education level, smoking, drinking, physical exercise, BMI, and chronic diseases.

## Data Availability

Data presented in this study are available from the corresponding author upon reasonable request. The data are not publicly available due to subjects’ privacy.

## Supplementary Materials

The following are available online at https://www.mdpi.com/article/10.3390/nu14204221/s1, Figure S1: Participant Flow Chart.
