# Supplementary material for: Psychiatric Symptoms and Frequency of Eating out among Commuters in Beijing: A Bidirectional Association?
Source: Nutrients. 2022 Oct 11;14(20):4221. doi: 10.3390/nu14204221 (PMC9609142; doi:10.3390/nu14204221)

Psychiatric Symptoms and Frequency of Eating Out among Commuters in Beijing: a Bidirectional Association? Ling Zhang et al. Online Supplementary Material.

Supplementary Figure S1. Participant Flow Chart according to the STROBE statement

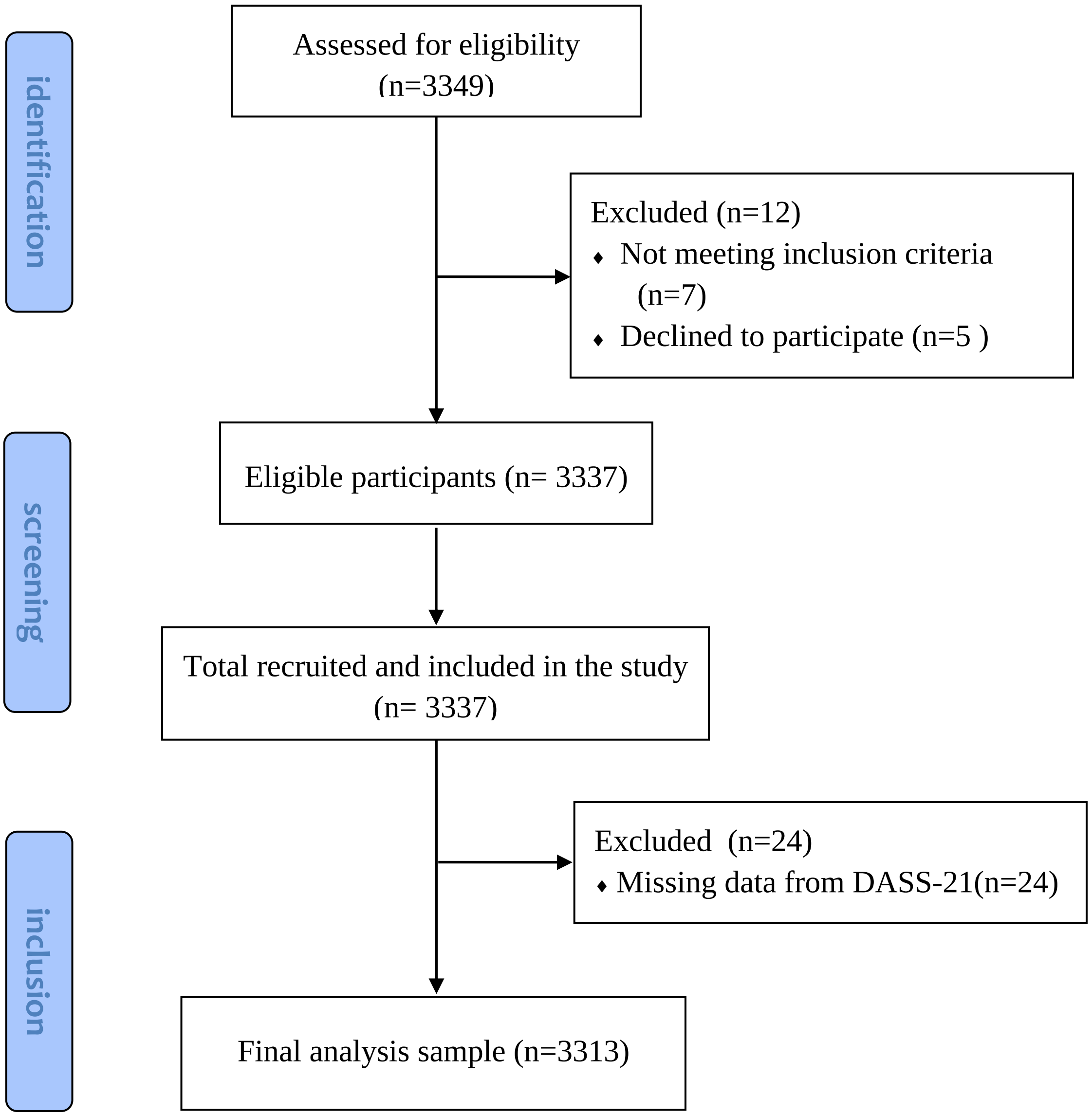

Supplement: Supplementary file 1 [file nutrients-14-04221-s001.zip › nutrients-1956889-supplementary.pdf]
